# Investigating the Effect of Steric Hindrance within CdS Single-Source Precursors on the Material Properties of AACVD and Spin-Coat-Deposited CdS Thin Films

**DOI:** 10.1021/acs.inorgchem.2c00616

**Published:** 2022-05-18

**Authors:** Mark A. Buckingham, Kane Norton, Paul D. McNaughter, George Whitehead, Inigo Vitorica-Yrezabal, Firoz Alam, Kristine Laws, David J. Lewis

**Affiliations:** † Department of Materials, The University of Manchester, Manchester M13 9PL, U.K.; ‡ Department of Chemistry, The University of Manchester, Manchester M13 9PL, U.K.; § Department of Chemistry, King’s College London, London SE1 1DB, U.K.

## Abstract

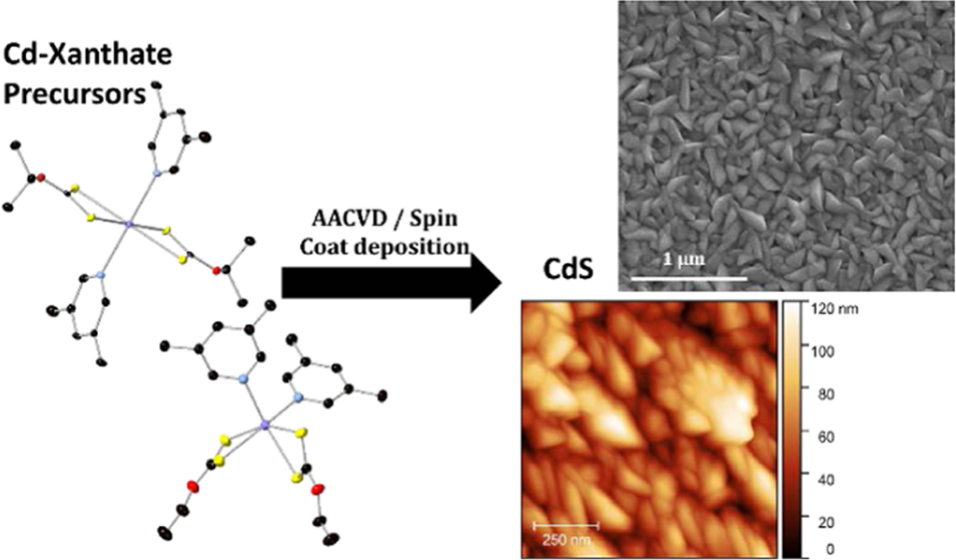

Cadmium sulfide (CdS) is an important semiconductor for electronic and photovoltaic applications, particularly when utilized as a thin film for window layers in CdTe solar cells. Deposition of thin-film CdS through the decomposition of single-source precursors is an attractive approach due to the facile, low-temperature, and rapid nature of this approach. Tailoring the precursor to affect the decomposition properties is commonly employed to tune desirable temperatures of decomposition. However, altering the precursor structure and the effect this has on the nature of the deposited material is an area far less commonly investigated. Here, we seek to investigate this by altering the ligands around the Cd metal center to increase the steric hindrance of the precursor and investigate the effect this has on the decomposition properties and the properties of deposited thin-film CdS from these precursors. For this, we report the synthesis of four CdS precursors with xanthate and pyridyl ligands ([Cd(*n*-ethyl xanthate)_2_(3-methyl pyridine)_2_] **[1]**, [Cd(*n*-ethyl xanthate)_2_(3,5-lutidine)_2_] **[2]**, [(Cd_2_(isopropyl xanthate)_4_(3-methyl pyridine)_2_)*_n_*] **[3]**, and [Cd(isopropyl xanthate)_2_(3,5-lutidine)_2_] **[4])**. These single-source precursors for CdS were fully characterized by elemental analysis, NMR spectroscopy, single-crystal X-ray diffraction (XRD), and thermogravimetric analysis. It was found that even with subtle alterations in the xanthate (*n*-ethyl to isopropyl) and pyridine (3-methyl and 3,5-dimethyl) ligands, a range of hexa-coordinate precursors were formed (two with *cis* configuration, one with trans configuration, and one as a one-dimensional (1D) polymer). These four precursors were then used in aerosol-assisted chemical vapor deposition (AACVD) and spin-coating experiments to deposit eight thin films of CdS, which were characterized by Raman spectroscopy, powder X-ray diffraction, and scanning electron microscopy. Comparative quantitative information concerning film thickness and surface roughness was also determined by atomic force microscopy. Finally, the optical properties of all thin films were characterized by ultraviolet–visible (UV–Vis) absorption spectroscopy, from which the band gap of each deposited film was determined to be commensurate with that of bulk CdS (*ca.* 2.4 eV).

## Abstract 2

Four single-source CdS precursors were synthesized based on a combination of xanthate- and pyridyl-derived ligands to investigate increasing the steric hindrance of the precursor. Two *cis*, one *trans*, and one 1D polymer complexes were developed. These precursors were then deposited as thin films through both spin coating and aerosol-assisted chemical vapor deposition techniques, and the morphology, film thickness, film surface roughness, particle size distribution, and band gap energy were assessed.

## Introduction

Thin-film semiconductors are important for energy applications such as photovoltaics,^1−3^ thermoelectrics,^4−6^ and electronic applications such as transistors.^7,8^ Homogenous thin-film semiconductors are attractive^8^ as they can achieve good electrical conductivities^9^ and low thermal conductivities^6^ and can provide high power-to-weight performances in solar cells.^1^ To date, there has been a wide range of methods reported to deposit thin-film semiconductors such as plasma-enhanced chemical vapor deposition (PECVD),^10,11^ chemical bath deposition (CBD),^12,13^ aerosol-assisted chemical vapor deposition (AACVD),^14,15^ metal–organic chemical vapor deposition (MOCVD),^16,17^ spin coating,^9,18^ and spray pyrolysis.^19−21^ This wide range of deposition techniques combined with the plethora of both metal oxides and chalcogenides that have been investigated has driven research in this area. Cadmium sulfide (CdS) is a binary II–VI inorganic semiconductor with a direct band gap (*E*_g_) of 2.4 eV.^14^ CdS has attracted attention in photovoltaic, photoelectrocatalysis, and electronic applications.^22−29^ CdS/CdTe heterojunction photovoltaic cells in particular have been of particular interest due to the high efficiency (up to 20%) in these devices.^27,30^ A number of processing routes have been reported for the synthesis of CdS thin films, but many still suffer from problematic features. For example, chemical bath deposition requires high concentrations (up to 1 M) of a Cd salt to deposit CdS as a thin film.^31^ Other techniques using elemental or ionic Cd can be used to deposit CdS; however, these require high temperatures (up to 500 °C).^32−34^ Both of these required conditions are unfavorable from a green chemistry perspective as the processes are either energy intensive or utilize large quantities of highly toxic Cd. Due to the high toxicity of cadmium, exposure should be limited as much as possible, using as little of the toxic material as possible.^35^ A lower-temperature synthetic method of forming CdS thin films could result in significant energy savings, particularly when considering a scaled-up synthesis.

Single-source molecular precursors are an excellent route toward both thin-film and nanoparticulate metal chalcogenides.^36^ Combining the metal–chalcogenide bond in the precursor itself prior to the decomposition–deposition step negates any side reactions between separate metal and chalcogenide sources and pre-reactions in the feed.^36^ Containing potentially toxic metals in a nonvolatile, nonpyrophoric, and air-stable precursor is also beneficial from a safety perspective.^35^ A range of metal–organic cadmium complexes have been reported for deposition of CdS, such as dithiobiurets,^37^ dithioacetylacetonate,^38^ and dithiophosphinato^39^ complexes. Perhaps the most utilized ligands for these complexes are based on dithiocarbamates.^40,41^ A recent review by Hogarth on metal dithiocarbamate complexes demonstrates the huge scope of these ligands to sequester almost every metal in many different oxidation states.^36^ Beyond these commonly used ligands, xanthate (dithiocarbonate, S_2_COR)-based complexes are very attractive as they form stable complexes with the target metal, similar to dithiocarbamates,^14,26,42^ and with clean decomposition to the metal sulfide also producing volatile side products, which do not contaminate the produced metal sulfide. Advantages of xanthate over dithiocarbamate complexes are the better atom economy^36^ and the significantly lower decomposition temperature; the latter is believed to arise from the facile Chugaev elimination that is thought to be responsible for the very clean decomposition.^36,43^ Tuning the structure of the precursor has previously been reported to have a significant effect on properties such as the decomposition temperature.^14,42^ This is typically achieved by significantly increasing the length of the R group in the S_2_COR ligand;^42,44^ however, these studies typically focus on the precursor rather than analyzing the deposited films.

To the best of our knowledge, there has not yet been an attempt to measure thin-film properties such as growth plane, morphology, and deposited film thickness and an attempt to determine if this can be related to the structure of the initial molecular precursor. We also set out here to undertake a direct comparison between two common deposition techniques (AACVD and spin coating) and ascertain if the properties of these deposited films could be related to the precursor over different deposition techniques. A direct comparison of nanostructured CdS thin films has been undertaken using two different deposition techniques (CBD and spray pyrolysis)^33^ with respect to the decomposition annealing temperature (which is high due to the nature of these two techniques). To address this knowledge gap, herein we report the synthesis of four single-source CdS precursors based on combinations of two xanthate ligands (*n*-ethyl and isopropyl) and two pyridine derivatives (3-methyl and 3,5-dimethyl). These ligands were selected as they were not expected to significantly alter the size of the molecular precursor, but this selection would allow us to investigate the effect of increasing steric hindrance of the molecular precursor and investigate if tuning this property in the precursor can also tune the material properties of the deposited thin films. These precursors were fully characterized for their structural and decomposition properties and deposited as thin films through AACVD and spin-coating methods. These films were characterized by Raman spectroscopy and powder X-ray diffraction (XRD), along with morphological analysis by scanning electron microscopy (SEM) and atomic force microscopy (AFM). Comparative quantitative information concerning film thickness and surface roughness was also determined by AFM analysis. The band gap of all of the deposited thin films was also determined through ultraviolet–visible (UV–Vis) spectroscopy.

## Experimental Section

### Chemicals

All chemicals were purchased from UK suppliers and used without further purification, unless specified. These were cadmium nitrate tetrahydrate (98%, Sigma-Aldrich), potassium ethyl xanthogenate (potassium ethyl xanthate, 96%, Sigma-Aldrich), *o*-isopropylxanthanthic acid potassium salt (potassium isopropyl xanthate, 96%, Sigma-Aldrich), 3-methyl pyridine (Fluorochem), and 3,5-lutidine (≥98%, Sigma-Aldrich).

### Note of Cd Toxicity

Cadmium is a highly toxic metal that is a suspected carcinogen according to the safety data sheet (SDS) of the Cd salt used in this report. Therefore, preparation using this salt and handling of the salt itself must be undertaken with extreme caution and the relevant protective equipment required for handling such a highly toxic and suspected carcinogenic metal.

### Instrumentation

NMR analysis was conducted on a Bruker, 400 MHz machine. Elemental analysis (EA) was performed on a Thermo Scientific Flash 2000 Organic Elemental Analyzer for CHN and S analyses and a Thermo Scientific iCAP 6000 series ICP Spectrometer for metal analysis. Thermogravimetric analysis (TGA) and differential scanning calorimetry (DSC) were simultaneously performed on a Mettler Toledo TGA/DSC1 STARe system, under a N_2_ atmosphere and a heat ramp rate of 10 °C min^–1^. EA, TGA, and DSC were all conducted by the microanalytical suite at the University of Manchester. Single-crystal XRD data for **[2]** and **[3]** were collected using a Rigaku FR-X DW diffractometer, equipped with a 4-circle AFC-11 RINC kappa goniometer and an Oxford Cryosystems Cryostream 800 plus, using Cu Kα (λ = 1.54184) radiation at a temperature of 100 K. XRD data for **[4]** were collected using an Agilent Supernova diffractometer, equipped with a 4-circle goniometer and an Oxford Cryosystems Cryostream 700, using Mo Kα (λ = 0.71073) radiation at a temperature of 100 K. All data were collected and reduced using Rigaku CrysAliPro v171.41. Single-crystal X-ray structures were solved using ShelXT, refined using ShelXL, and implemented through Olex2 v1.5. Grazing incidence powder XRD (GI-pXRD) was conducted on a Philips X’pert modular powder diffractometer, using Kα radiation (Kα 1.540598). Data were collected with a fixed incidence angle of 1° and a 2θ scan range of 5–70° at a ramp of 0.02°. Raman spectroscopy was performed on a Horiba LabRAM instrument using a 488 nm wavelength laser. AFM was performed using a JPK NanoWizard 4 AFM with TESPA V2 AFM tips. Samples were fixed in place using double-sided tape on a glass microscope slide. Films were scored using a scalpel to measure the thickness. Scanning electron microscopy (SEM) analysis was performed on an FEI Quanta 650 FEG operating at 20 kV. Optical measurements were recorded on a Shimadzu UV-1800 in the wavelength range of 1100–300 nm.

### Synthesis of the CdS Precursor Complexes

All complexes were synthesized from an adapted literature procedure.^14^ The solids of cadmium nitrate (*ca.* 1 g, 3 mmol) and the xanthate of choice (either ethyl or isopropyl, *ca.* 1 g, 6.2 mmol) were combined in a round-bottom flask containing a stirrer bar and placed under an Ar atmosphere. To this, dry tetrahydrofuran (THF) (50 mL) was added, followed immediately by the pyridine derivative of choice (either 3-methyl pyridine or 3,5-lutidine, *ca.* 600 μL, 6.2 mmol). Once all components had been added, the solution was stirred and left overnight (typically *ca.* 18 h). The formed precipitate (KNO_3_) was filtered and washed with THF. The solution containing the product was then evaporated to dryness under vacuum and recrystallized in acetone. Yields of these precursors were *ca.* 60–80%.

### Analysis of the CdS Precursor Complexes

**Figure 1:**
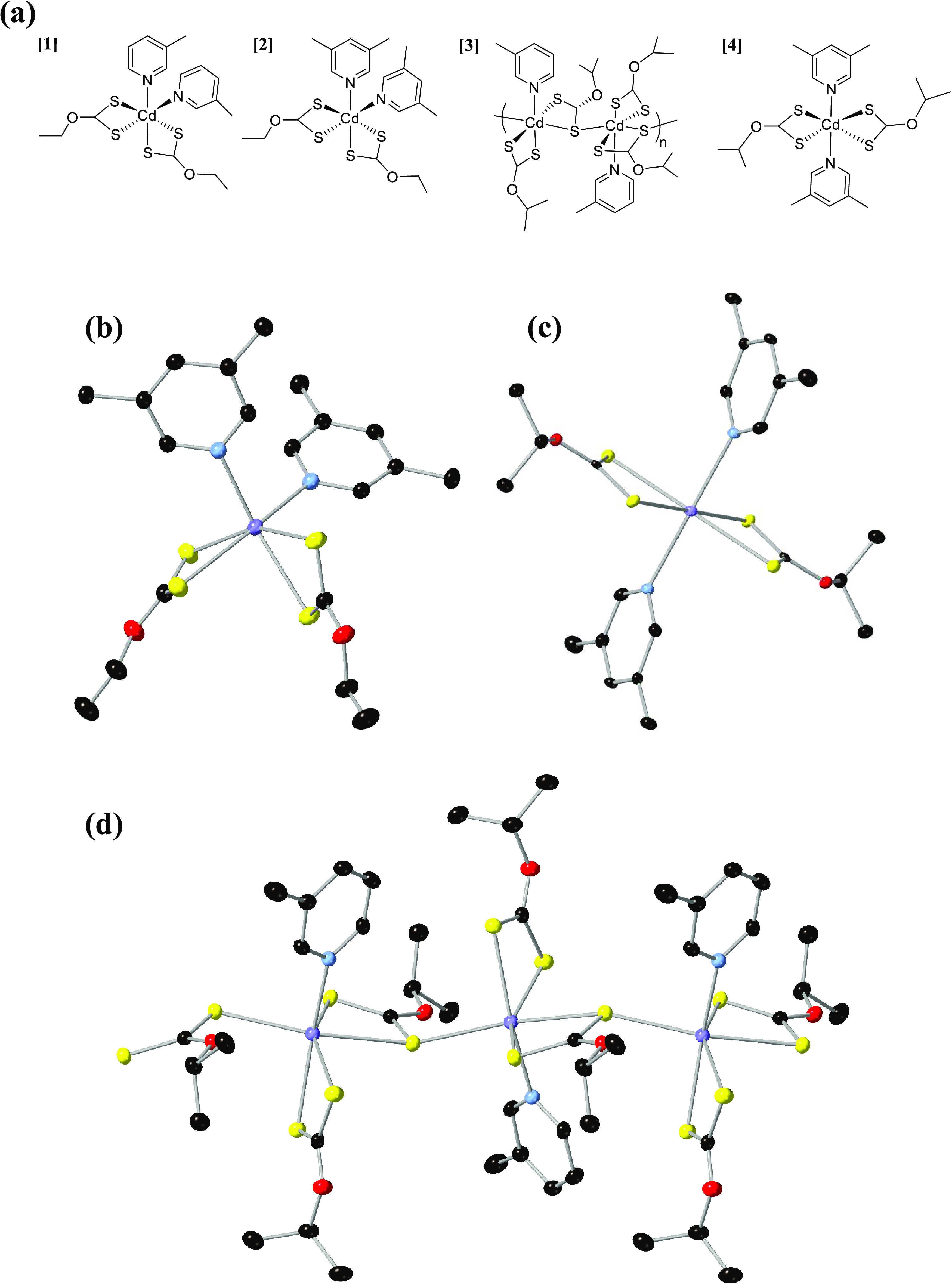
Figure showing (a) chemical structures of the four synthesized precursors ([Cd(*n*-ethyl xanthate)_2_(3-methyl pyridine)_2_] **[1]**, [Cd(*n*-ethyl xanthate)_2_(3,5-lutidine)_2_] **[2]**, [(Cd_2_(isopropyl xanthate)_4_(3-methyl pyridine)_2_)*_n_*] **[3]**, and [Cd(isopropyl xanthate)_2_(3,5-lutidine)_2_] **[4]**). Also shown are (b), (c), and (d) the crystal structures of **[2]**, **[4]**, and **[3]**, respectively (cadmium = purple, sulfur = yellow, oxygen = red, nitrogen = blue, and carbon = black. Hydrogen atoms and solvent molecules are omitted for clarity). Full information on bond angles, lengths, and crystal determination parameters can be found in the supporting information (Figures S2–S4 and Table S1).

Complex **[1]** (Figure 1) has been previously characterized by NMR, EA, and single-crystal XRD.^14^ The successful synthesis of this complex here was confirmed by ^1^H and ^13^C NMR. Main IR peaks (cm^–1^): 645, 702, 787, 1029, 1119, 1181, 1438, 1581, 1603, and 2983.

#### Analysis of Complex **[2]** (Figure 1) Found

Elemental analysis found (calculated for CdC_20_H_28_N_2_O_2_S_4_ (in %)); C: 42.3 (42.2), H: 4.9 (4.9), N: 5.2 (4.9), S: 22.3 (22.6), Cd: 19.6 (19.8). ^1^H NMR (400 MHz, DMSO): δ 8.21 (s, 4 H), 7.43 (s, 2 H), 4.34 (q, 4 H), 2.25 (s, 12 H), 1.30 (t, 6 H). ^13^C NMR: ^13^C NMR (101 MHz, DMSO, {^1^H}): δ 229.76, 147.42, 137.64, 132.94, 72.89, 18.20, 14.46. Main IR peaks (cm^–1^): 649, 700, 744, 816, 858, 936, 1031, 1115, 1139, 1175, 1356, 1379, 1384, 1435, 1472, 1594, 2977.

#### Analysis of Complex **[3]** (Figure 1) Found

Elemental analysis found (calculated for CdC_14_H_21_N_1_O_2_S_4_ (in %)); C 35.4 (35.1), H 4.4 (4.4), N 3.2 (2.9), S 26.7 (26.9), Cd 23.6 (23.5). ^1^H (400 MHz, DMSO): δ 8.41 (dd, 1 H), 8.38 (dd, 1 H), 7.62 (d, 1 H), 7.30 (dd, 1 H), 5.12 (sept, 2H), 2.29 (s, 3 H), 1.30 (d. 12 H). ^13^C NMR (101 MHz, DMSO, {^1^H}): δ 228.78, 150.24, 147.13, 137.16, 133.58, 123.89, 81.05, 21.75, 18.40. Main IR peaks (cm^–1^): 647, 699, 812, 820, 901, 1013, 1088, 1140, 1192, 1215, 1324, 1367, 1420, 1459, 1483, 1580, 2872, 2930, 2976.

#### Analysis of Complex **[4]** (Figure 1) Found

Elemental analysis found (calculated for CdC_20_H_28_N_2_O_2_S_4_ (in %)); C: 44.0 (44.2), H: 5.4 (5.4), N: 4.7 (4.7), S: 21.6 (21.5), Cd: 18.5 (18.8). ^1^H NMR (400 MHz, DMSO): δ 8.21 (d, 4 H), 7.43 (s, 2 H), 5.13 (sept, 2 H), 2.25(s, 12 H), 1.29 (d, 12 H). ^13^C NMR (101 MHz, DMSO, {^1^H}): δ 228.55, 147.44, 137.56, 132.89, 80.77, 21.74, 18.20. Main IR peaks (cm^–1^): 2983, 1591, 1458, 1378, 1196, 1141, 1085, 1031, 936, 908, 860, 815, 740, 715, 701, 649.

IR spectra are shown in Figure S1.

### AACVD Deposition

Prior to any deposition by either AACVD or spin coating, glass substrates were cleaned by ultrasonication in acetone for 10 min, followed by ultrasonication in isopropanol for a further 10 min.

CdS thin films were deposited on clean glass substrates (1.5 cm × 3 cm) using aerosol-assisted chemical vapor deposition. The CdS precursor (0.2 g) was dissolved in 20 mL of THF, and this solution was held in a two-neck round-bottom flask with an inlet of Ar flowing at *ca.* 250 sccm to transport the aerosol, controlled by a Platon flow gauge. The second neck of the round-bottom flask was used as an outlet for the aerosol, which was connected to the reactor tube by a piece of reinforced rubber tubing. The quartz reactor flask containing five glass substrates was placed inside a Carbolite tube furnace, which was maintained at 250 °C prior to any aerosol transport. The aerosol was generated using a piezoelectric modulator of a PIFCO ultrasonic humidifier (model 1077), and the aerosol was generated for 60 min.

### Spin-Coating Deposition

A solution of the CdS precursor (0.1 g in 1 mL of THF) was deposited (200 μL) on a pre-cleaned glass substrate (1.5 cm × 3 cm). This was subjected to spin coating at 1500 rpm for 30 s (Ossila spin coater). The subsequent glass substrate with the thin film of the precursor solution was then placed in the quartz reaction vessel in a Carbolite furnace and decomposed at 250 °C for 1 h under an Ar atmosphere.

## Results and Discussion

### Synthesis of the CdS Precursor Complexes

Four CdS precursors were synthesized based on a combination of two pyridine derivates (3-methyl pyridine and 3,5-lutidine) and two xanthate ligands (*n*-ethyl xanthate or isopropyl xanthate). These ligands were selected as they directly investigate the steric hindrance of the coordination around the metal center, without significantly increasing the size of the precursor structure. The chemical structures of the four synthesized precursors are shown in Figure 1a. Structure **[1]** has been previously reported with a *cis*, *cis*, *cis*-configuration,^14^ consistent with the underived pyridine complex.^26^ Here, substituting the 3-methyl pyridine for 3,5-lutidine yielded precursor **[2]**, which was also found to form the *cis*, *cis*, *cis*-configuration, as also evidenced by the single-crystal X-ray structure (Figure 1b). Formation of precursor **[4]** with the most substituted 3,5-lutidine and isopropyl xanthate ligands was found to produce the *trans* equivalent (Figure 1a,c), which is consistent with a previous Cd–xanthate complex containing the *n*-butyl xanthate.^45^ Precursor **[3]** was found to be more complicated. After synthesis, an oil was often formed; once this was dried, the NMR, EA, and decomposition products of TGA (discussed later) were consistent with a monopyridine complex. Crystals were eventually grown of this complex and the crystal structure was determined. This analysis showed a one-dimensional (1D) polymeric complex had been formed, which was also a hexacoordinate complex with a bridging Cd–S–Cd bond from one of the xanthate ligands (Figure 1a). The differences between these precursor structures yield important insight into the steric effects. The *cis*, *cis*, *cis*-configuration is clearly the most favorable, with the bulk of these complexes forming this structure.^14,26^ Significant steric hindrance is required to yield the analogous *trans* complex, with hindrance on both the pyridine and xanthate ligands. In the absence of this significant steric hindrance, the complex formed a 1D polymer, further demonstrating that formation of the *trans* complex is significantly unfavorable.

### Decomposition of the CdS Precursors

**Figure 2:**
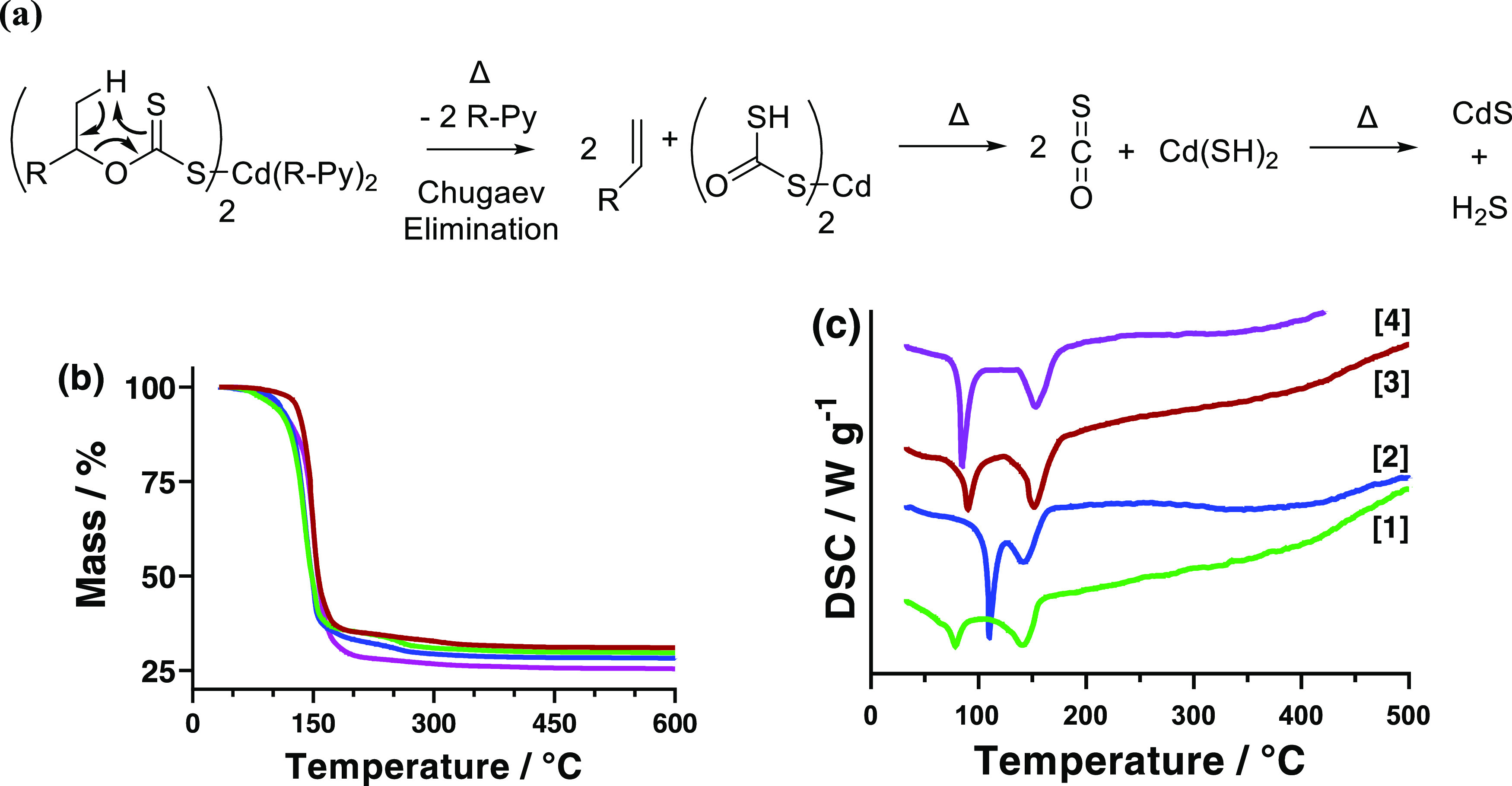
Figure showing the (a) proposed decomposition pathway of the CdS precursors.^14^ Also shown are the (b) thermogravimetric analysis (TGA) and (c) differential scanning calorimetry (DSC) of the four investigated precursors where **[1]** (green), **[2]** (blue), **[3]** (red), and **[4]** (purple) are all shown. The comparable DSC traces are shown in Figure S5.

The decomposition behavior of the four synthesized precursors was next investigated. Figure 2 shows both the (b) thermogravimetric analysis (TGA) and (c) differential scanning calorimetry (DSC) of the four investigated precursors. The TGA plot shows that complexes **[1]**, **[2]**, and **[4]** all observe a gradual initial decomposition, followed by a rapid decomposition to initially leave Cd(SH)_2_, followed by a slow decomposition and condensation of H_2_S to the final CdS product, in line with previous results.^14^ Complex **[3]** appears to differ from the other precursors during the initial decomposition (likely caused by the significantly different nature of the precursor as a 1D polymer rather than single-metal molecules), instead of following a single major decomposition at around the same temperature (*ca.* 140–150 °C). It has been previously proposed that the labile pyridine adducts are initially lost in the decomposition in tandem with the alkene from the xanthate,^14^ in line with the proposed Chugaev elimination reaction (Figure 2a).

To further analyze the decomposition properties of the four precursors, DSC was also used, and the results are shown in Figure 2c. The DSC analysis for all precursors showed two endothermic peaks at different temperatures. The second of these can be attributed to the decomposition of the precursors as they also all occur within the 140–155 °C range. Therefore, the initial endothermic peak can be attributed to the melting of the solid Cd complex. These melting points were found to be more variable between 80 and 110 °C. Surprisingly, the two six-coordinate *cis*, *cis*, *cis*-configuration complexes observed the lowest (**[1]**) and highest (**[2]**) melting points (a full table of exact values of both melting and decomposition temperatures is shown in Table S2). It is also noteworthy that the melting of the two 3-methyl pyridine-containing complexes (**[1]** and **[3]**) are significantly less endothermic than the two 3,5-lutidine-containing complexes (**[2]** and **[4]**).

### Thin-Film Preparation and Characterization by pXRD and Raman Spectroscopy

Having synthesized and characterized the decomposition of the four synthesized CdS precursors, these complexes were next deposited as thin films on glass substrates. As the aim of this report was to assess the characteristics of the deposited CdS based on the input precursor, two different deposition techniques were used, with the same deposition conditions for both (250 °C for 1 h under an Ar atmosphere). Aerosol-assisted chemical vapor deposition (AACVD) is a technique that has become increasingly investigated as a scalable alternative to other chemical vapor deposition methods.^46−48^ Here, we also investigated spin coating as a deposition technique for thin-film preparation.^49^ Using these two techniques on all four precursors, eight films were prepared (all shown in Figure S6). These films were all characterized by powder X-ray diffraction (pXRD) and Raman spectroscopy.

**Figure 3:**
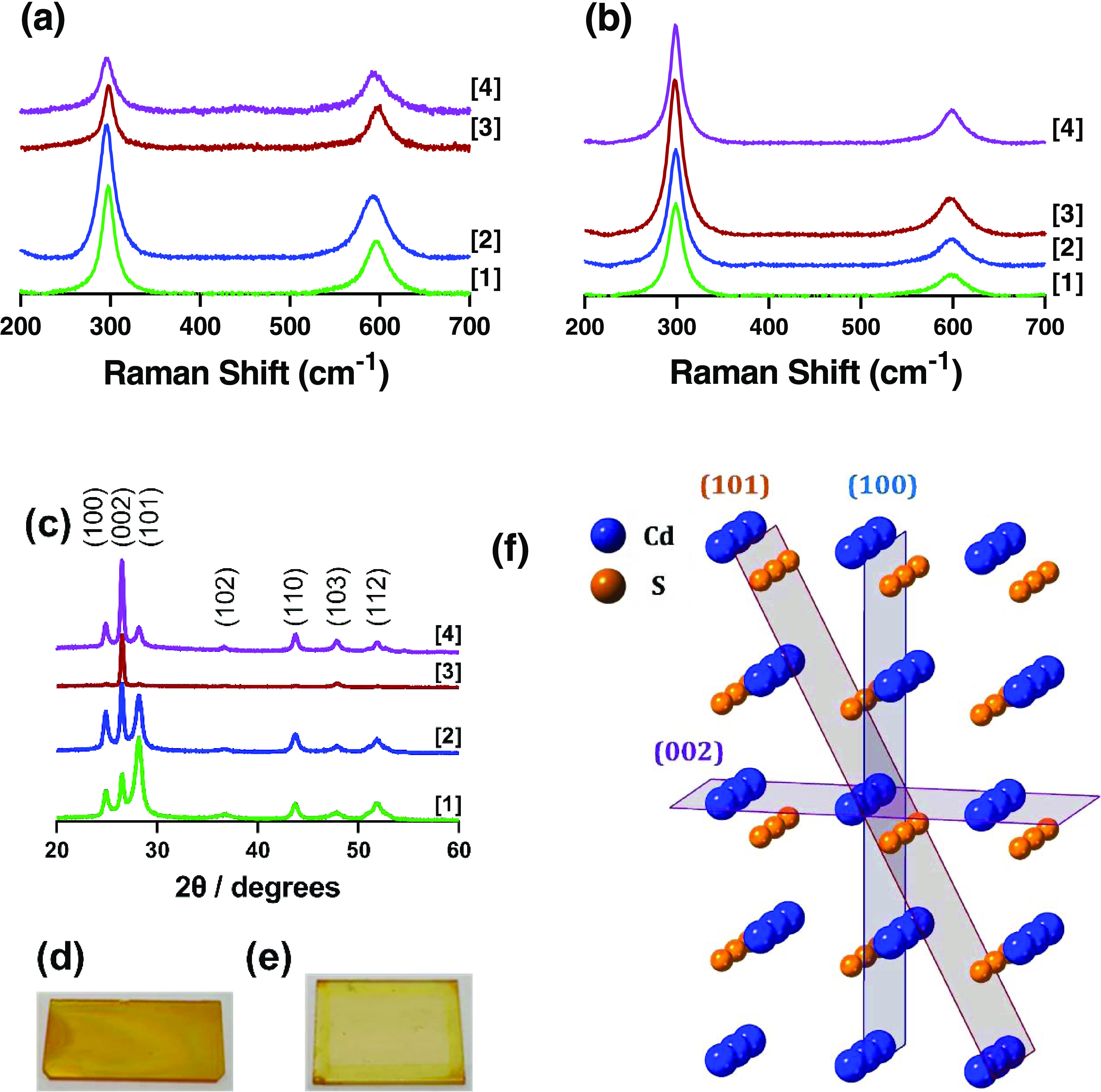
Figure showing the Raman spectra of (a) AACVD and (b) spin-coat-deposited thin films. (c) pXRD of AACVD-deposited thin films, with the planes indicated above the patterns. Also shown are representative films deposited by (d) AACVD, (e) spin coating, and (f) the crystal structure for hexagonal CdS, with the three main planes shown and indicated.

Raman spectroscopy was used to characterize the deposited films. Raman spectra were recorded using a 488 nm wavelength laser with a 50× magnification. Both AACVD- (Figure 3a) and spin-coat (Figure 3b)-deposited films displayed two peaks centered at *ca.* 300 and 600 cm^–1^ (exact values are shown in Table S3). We assign these to the two longitudinal optical (LO) modes of hexagonal CdS.^50,51^ The significantly greater 1LO mode over the 2LO mode is consistent with bulk, rather than nanocrystalline CdS.^14,50^ Representative films deposited by AACVD (Figure 3d) and spin coating (Figure 3e) for complex **[2]** are also shown.

Having ascertained that bulk CdS had been deposited across all eight films, pXRD patterns were next measured for both the AACVD (Figure 3c) and spin-coated (Figure S7) films. No appreciable pattern could be obtained from the spin-coat-deposited materials due to the extremely thin nature of the films (*vide infra*). The AACVD-deposited films showed that excellent crystallinity was achieved for hexagonal CdS. The preferred orientation of the films with respect to the substrate, however, appeared to be dependent on the precursor initially used. Precursor **[1]** favored the (101) plane, precursor **[2]** showed little preference and obtained almost equal intensity for both (002) and (101) planes, and both **[3]** and **[4]** showed a large preference for the (002) plane (crystalline planes are visually represented for the crystal structure as shown in Figure 3f for the three main planes (100, 101, 002) found in these deposited films). This was repeated with precursor **[3]**, which again showed a strong preference for the (002) growth plane (not shown). This therefore allows us to tentatively suggest that the growth plane of hexagonal CdS can be tailored by judicious choice of the precursor structure. However, it should be noted that this is under the specific conditions employed here. Temperature, deposition time, precursor concentration, and solution volume are all factors that are also known to affect the properties of the deposited materials,^46,52^ and a previous deposition using precursor **[1]** at a 2.5× higher concentration, higher and lower temperature, and a different AACVD setup obtained a favorability for the (002) plane.^14^ This will be the subject of future and more rigorous study, as the ability to tailor film growth in a particular plane could have significant importance for catalytic applications of CdS. For example, metal nanoparticles have been demonstrated to have a significantly different catalytic activity for both different planes^53,54^ and sizes,^55^ with the input (pre-catalyst) material changing under *operando* conditions.^56−59^

### SEM Analysis

**Figure 4:**
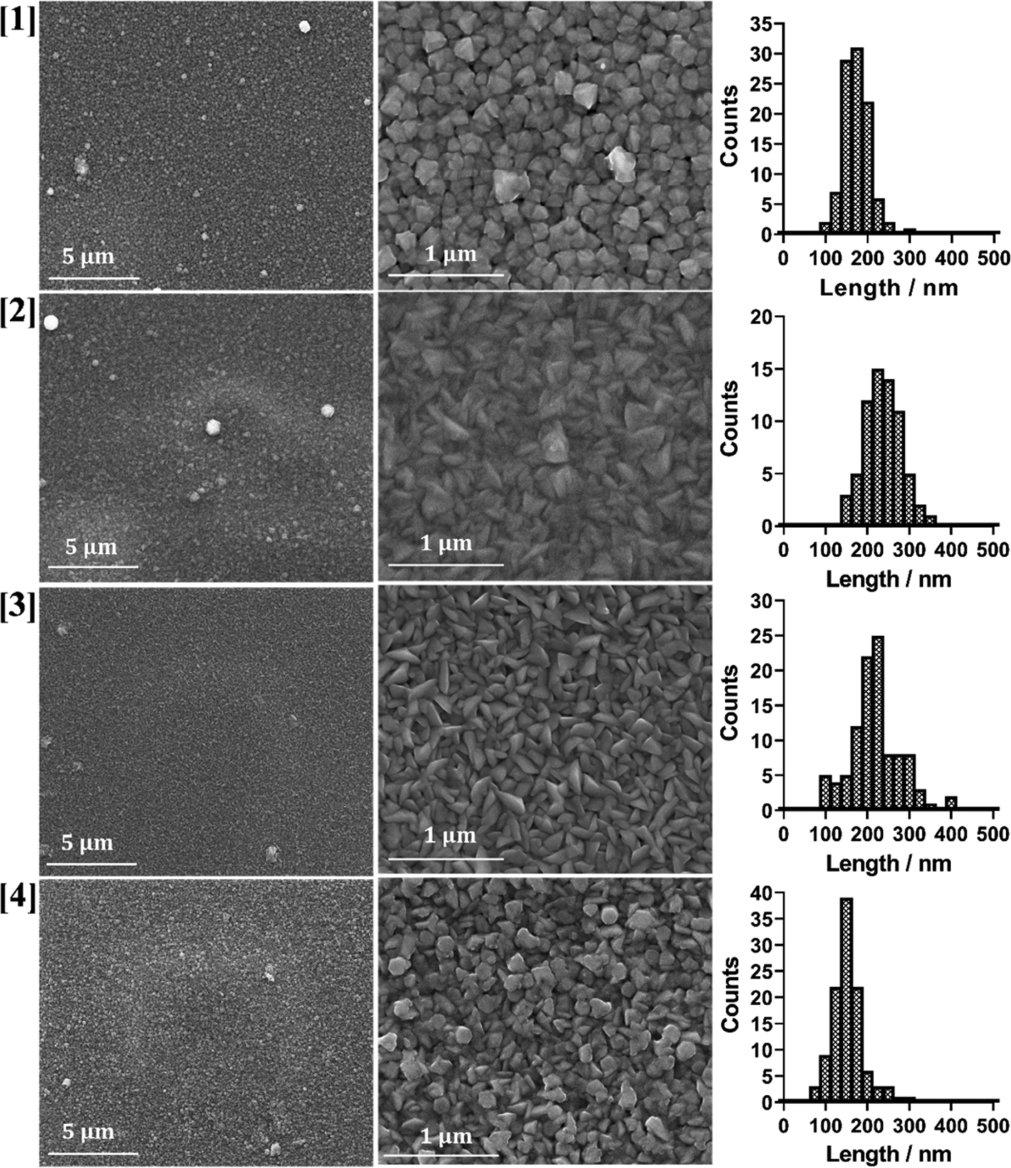
Figure showing representative SEM (secondary electron) images of the AACVD-deposited thin films at two different magnifications (first column 8000×, second column 50,000×) where rows 1, 2, 3, and 4 represent precursor **[1]**, **[2]**, **[3]**, and **[4]**, respectively.

Having characterized the CdS films for their crystallinity and favored growth plane, the morphology was next assessed. For this, scanning electron microscopy (SEM) was employed. Representative SEM images of the AACVD-deposited films show good coverage and relatively uniform crystallite sizes across the film, with a few larger particles dispersed (first column, Figure 4). Higher-magnification images of these films show different morphologies of the grown particles (second column, Figure 4). The thin films grown from precursors **[1]** and **[4]** produce more rounded particles and thin films grown from precursors **[2]** and **[3]** show more sharp-edged particles. With respect to the spin-coat-deposited films (Figure S8), SEM also shows that homogenous thin films are formed. However, the sizes of the particles are significantly smaller than those grown by AACVD (further discussed in the next section with AFM analysis); these are not able to be detected with the SEM instrument employed here. The higher-magnification images showed good distribution of particles across the surface. This allowed quantitative analysis of the size distribution of the deposited particles. Measuring a number of randomly selected particles along the longest axis of the particles, histograms of particle size distribution could be determined and are shown in Figure 4 (each row represents a different precursor). It was found that the two precursors that produced the sharp-edged particles (**[2]** and **[3]**) formed larger particles on average and the precursors that formed the more spherical particles (**[1]** and **[4]**) produced smaller particles on average. However, this cannot be translated to trends concerning the steric hindrance of the initial precursors or the preferred growth plane, as shown in the pXRD patterns.

### AFM Analysis

**Figure 5:**
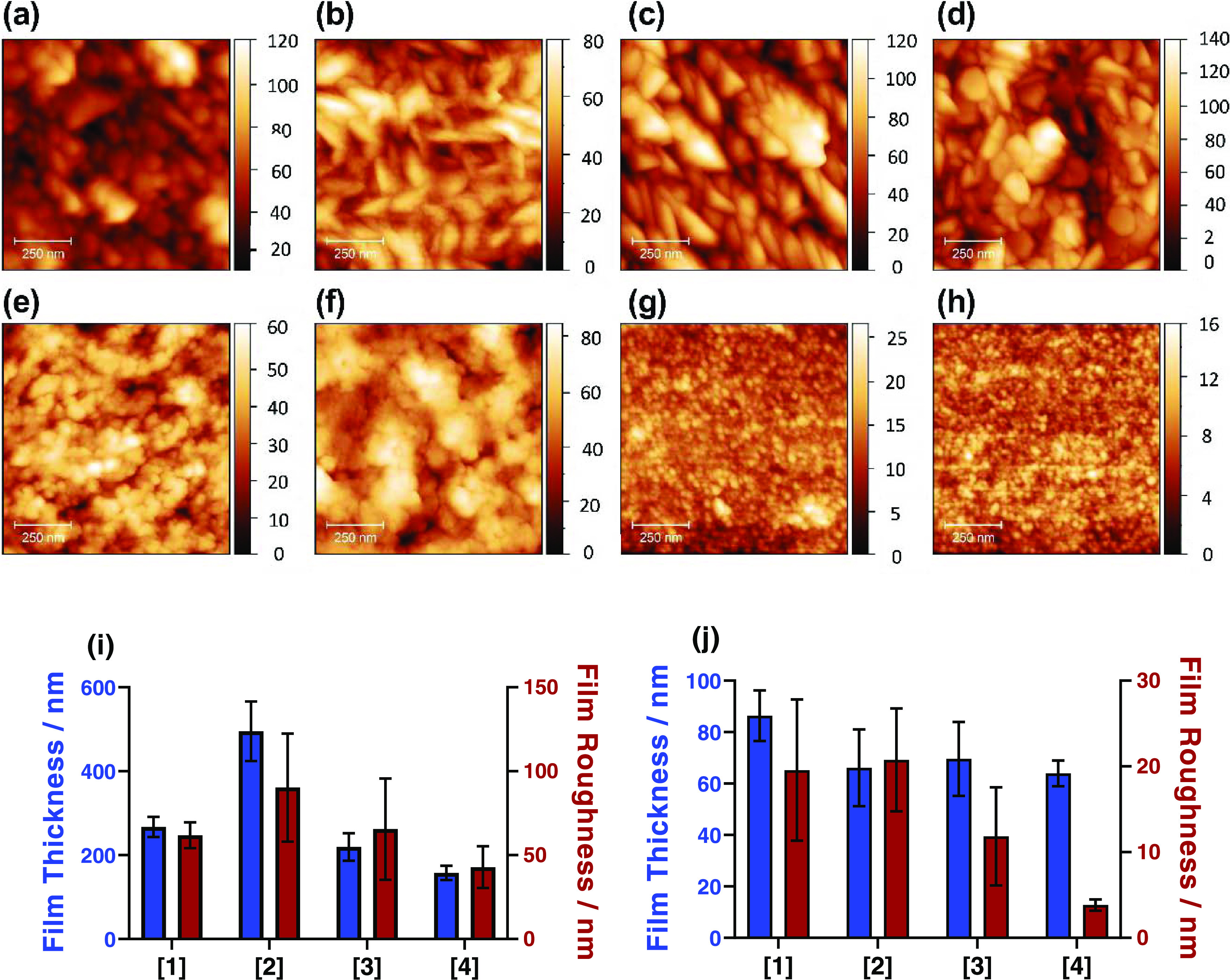
Figure showing (a–h) AFM images of the morphology of the thin films deposited by precursors (a, e) **[1]**, (b, f) **[2]**, (c, g) **[3]**, and (d, h) **[4]**, where (a–d) are deposited by AACVD and (e–h) are deposited by spin-coating deposition techniques. The scale bars to the right of each image are in nm. (i & j) show the measured film thickness and film surface roughness measured from AFM profiles (examples are shown in Figures S4 and S5), where (i) is for the AACVD deposited films and (j) is for the spin coat deposited films.

With the morphology of the produced films characterized at the macroscale, the surface of the films was probed at the nanoscale by atomic force microscopy (AFM). AFM analysis also yields further insight into the films and quantitative data can be obtained regarding both film thickness (as long as an edge-plane is observed) and surface roughness. Initially, the thickness of the deposited films was measured. For this, trenches in the film were made with a scalpel to obtain a direct path to the glass substrate these films were deposited on. The AFM probe was then scanned over the film, along with the edge of the trench allowing the direct determination of film thickness. Figure 5i,j shows the quantitative data obtained from this analysis with (i) representing AACVD-deposited films and (j) representing spin-coat-deposited films (both left *y*-axis). The spin-coat films are all of an equivalent (within error) thickness between 66 ± 16 and 86 ± 10 nm; this extremely thin nature of these films explains why the pXRD analysis did not detect any appreciable CdS signal. The AACVD-deposited films were observed to be significantly thicker and more variable, with **[4]** being the thinnest at *ca.* 160 ± 17 nm and **[2]** being the thickest at *ca.* 500 ± 71 nm (a full table of data for film thickness and roughness and representative images and profiles used in the thickness analysis in Table S4 is shown in Figures S9 and S10).

The surface roughness of these films can also be characterized using the same analysis; measuring only the CdS on the same edge-plane graphs, it is possible to obtain a surface roughness for the eight films. Figure 5i,j also shows the measured surface roughness (both right *y*-axis) of the eight films with (i) representing AACVD-deposited films and (j) representing spin-coat-deposited films. Films deposited by precursor **[4]** whether by AACVD or spin-coating produce homogenous films with low roughness. Whereas those films deposited by precursors **[2]** and **[3]** observe surfaces with high roughness for both AACVD and spin-coating deposition techniques. Films deposited by precursor **[1]** observe a low roughness when deposited by AACVD but a high surface roughness when deposited by spin coating.

Next, AFM was used to measure the surface topology of a pristine area of the CdS films to determine the nanoscale morphology and surface roughness (Figure 5a–h). This analysis (at lower length scales) indicates that the particles found for the AACVD-deposited films by SEM appear to consist of agglomerated, smaller particles. This combination of smaller particles is presumably the cause of the irregularly shaped particles observed with SEM (Figure 4). The difference in morphology between AFM and SEM analysis is due to the difference in technique: AFM is a direct surface topology technique, while secondary electron SEM at high electron voltage (20 keV) has an electron escape depth below the surface.^60^ Despite this, the morphology observed by AFM analysis matches that observed by SEM, where **[1]** and **[4]** and **[2]** and **[3]** share similar morphologies. AFM analysis of the spin-coat-deposited films also matches the SEM images obtained, with the films deposited from precursors **[1]** and **[2]** found to have similar morphologies of slightly larger particles conglomerating to a homogenous film and the films deposited from precursors **[3]** and **[4]** show equivalent morphology of small nanoparticles (which could not be detected by SEM). This is consistent with the substitution of the *n*-ethyl xanthate (**[1]** and **[2]**) to the isopropyl xanthate (**[3]** and **[4]**) increasing the steric hindrance of the precursor. However, this cannot necessarily be directly related to the alteration of the precursor structure.

### Optical Analysis of the Films

**Figure 6:**
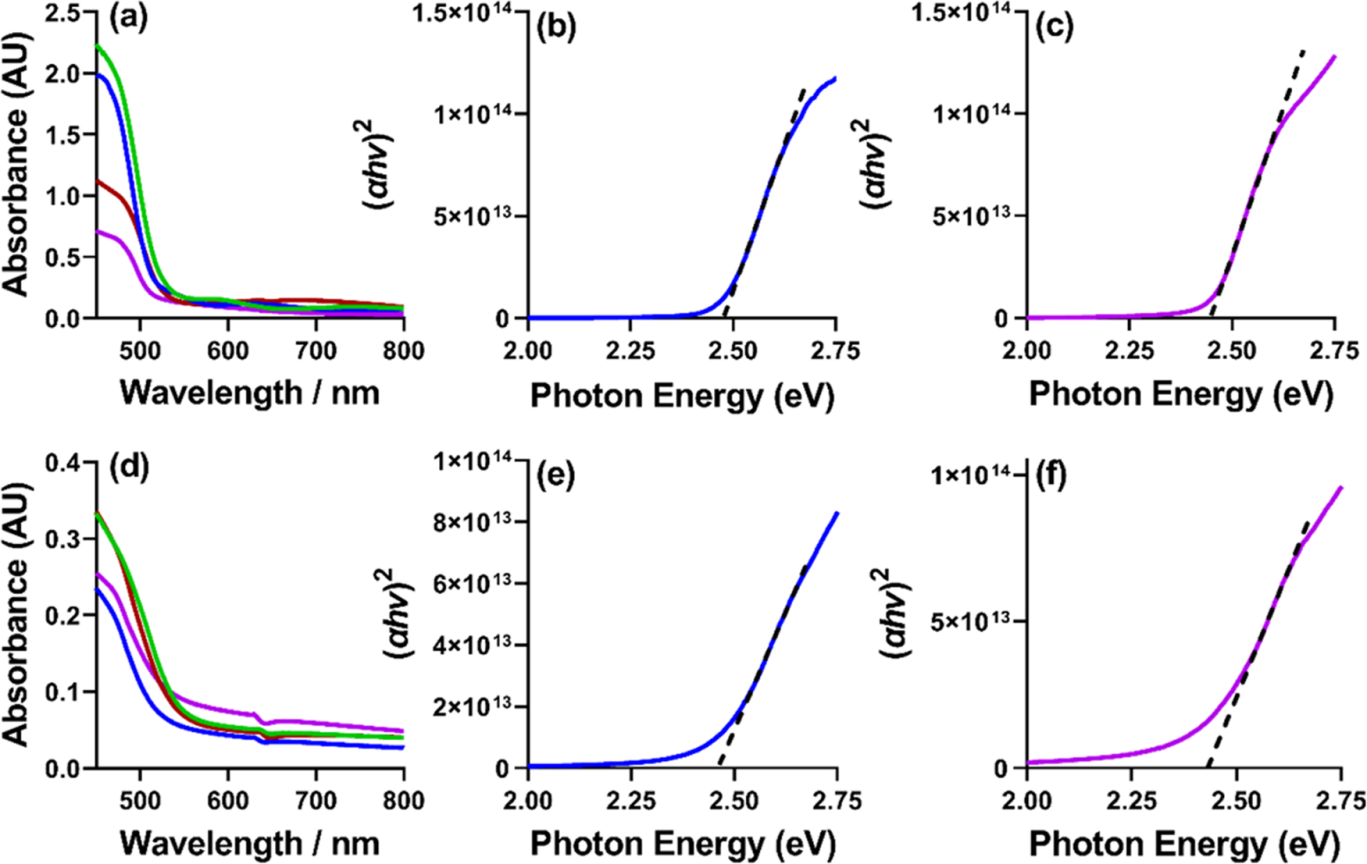
UV–vis absorption spectra of (a) AACVD-deposited thin films and (d) spin-coat-deposited thin films where films deposited by precursor **[1]** are shown in green, precursor **[2]** in blue, precursor **[3]** in red, and precursor **[4]** in purple. (b, c, e, f) Tauc plots determined from the (b, c) AACVD and (e, f) spin-coat-deposited films for precursors (b, e) **[2]** and (c, f) **[4]**; the other Tauc plots can be found in Figure S11.

The optical properties of the produced thin films were measured. Figure 6 shows the UV–Vis absorption spectra for (a) the AACVD and (b) the spin-coat-deposited films. All spectra observe an absorption between 550 and 500 nm. Using the Beer–Lambert law and the film thickness values obtained in the AFM analysis, Tauc plots can be obtained from these spectra, where Figure 6 shows the AACVD and spin-coated thin films deposited by precursors **[2]** (Figure 6b,e) and **[4]** (Figure 6c,f), respectively (the other film Tauc plots are shown in Figure S11, with a table of values in Table S5). All eight of the CdS thin films were found to observe a band gap within the range of 2.39–2.48 eV, which is in agreement with the reported band gap of bulk CdS (2.4 eV), demonstrating that all of these films have optical properties commensurate with the capture of significant amounts of solar flux for photovoltaic or absorption of light for photocatalytic applications.

## Conclusions

In this report, we have investigated the effect of increasing steric hindrance of the coordinating ligands of four xanthate-based CdS single-source precursors and whether this could influence the properties of the deposited CdS materials. This was achieved by substituting the *n*-ethyl to an isopropyl xanthate and a 3-methyl to a 3,5-dimethyl pyridine co-ligand. The subsequent thermal decomposition and deposition properties toward deposition of CdS thin films were investigated. This was achieved using two different methods (AACVD and spin coating) under equivalent decomposition conditions (with respect to time and temperature). The deposited thin films were characterized by pXRD and Raman spectroscopy, which determine that CdS was deposited in each case and different precursor structures have the potential to favorably deposit different growth planes of CdS. The morphology of these films was assessed by SEM and AFM analysis, which showed that different morphology of CdS was deposited, in line with different favored growth planes determined by pXRD. AFM analysis was also employed to measure the thickness and surface roughness of the investigated films, which showed significant differences in film thickness (between *ca.* 150 to 500 nm for AACVD and *ca.* 65–85 nm for spin-coat-deposited films) and roughness (*ca.* 40–90 nm for AACVD and *ca.* 4–20 nm for spin-coat-deposited films). Finally, the optical properties of the CdS thin films were investigated where all eight films were found to observe band gap energies in line with bulk CdS (*ca.* 2.4 eV), demonstrating the utility of the employed single-source precursors and the employed deposition techniques to desirable thin films of CdS.

## Supporting Information Available

Data on the crystallography of the precursors; tables of data on melting and decomposition, Raman spectroscopy, AFM analysis, and band gaps from UV–vis analysis; images of the deposited thin films; SEM images of the spin-coat samples; pXRD patterns of spin-coat-deposited films; and AFM analysis on film thickness (PDF)

## Accession Codes

CCDC 2152506–2152508 contain the supplementary crystallographic data for this paper. These data can be obtained free of charge via www.ccdc.cam.ac.uk/data_request/cif, or by emailing data_request@ccdc.cam.ac.uk, or by contacting The Cambridge Crystallographic Data Centre, 12 Union Road, Cambridge CB2 1EZ, UK; fax: +44 1223 336033.
