# Supplementary material for: Investigating the Effect of Steric Hindrance within CdS Single-Source Precursors on the Material Properties of AACVD and Spin-Coat-Deposited CdS Thin Films
Source: Inorg Chem. 2022 May 18;61(21):8206–16. doi: 10.1021/acs.inorgchem.2c00616 (PMC9157504; doi:10.1021/acs.inorgchem.2c00616)
Supplement: Supplementary file 1 — ic2c00616_si_001.pdf [file ic2c00616_si_001.pdf]

Supporting Information for:

Investigating the effect of steric hindrance within CdS single-source precursors on the material properties of AACVD and spin coat-deposited CdS thin films

Mark A. Buckingham,[a,*] Kane Norton,[a] Paul D. McNaughter,[b] George Whitehead,[b] Inigo Vitorica-Yrezabal,[b] Firoz Alam,[b] Kristine Laws[c] and David J. Lewis[a,*]

[a] Department of Materials, The University of Manchester, Manchester, M13 9PL, UK.

[b] Department of Chemistry, The University of Manchester, Manchester, M13 9PL, UK.

[c] Department of Chemistry, King's College London, London, SE1 1DB, UK.

* Corresponding authors: Mark A. Buckingham mark.buckingham@manchester.ac.uk and David J. Lewis, david.lewis-4@manchester.ac.uk

Contents

Figure S1 – IR Spectra of the four CdS precursors

Figure S2 – S4: Labelled crystal structures of **[1]**, **[2]** and **[3]** with selected bond distances and angles.

Table S1: Table of data for crystallography

Figure S5: Figure of comparative DSC

Table S2: Table of data for melting and decomposition measured by DSC

Figure S6: Images of the AACVD and spin coating deposited CdS thin films

Table S3: Table of data for Raman Spectroscopy

Figure S7: pXRD patterns of the spin coat deposited thin films

Figure S8: SEM images of the spin coat deposited thin films

Table S4: Table of data for AFM analysis

Figure S9 and S10: Representative images and analysis for AFM thickness determination

Figure S11: Tauc plots for **[1]** and **[3]**

Table S5: Table of data for Tauc analysis

IR Spectroscopy

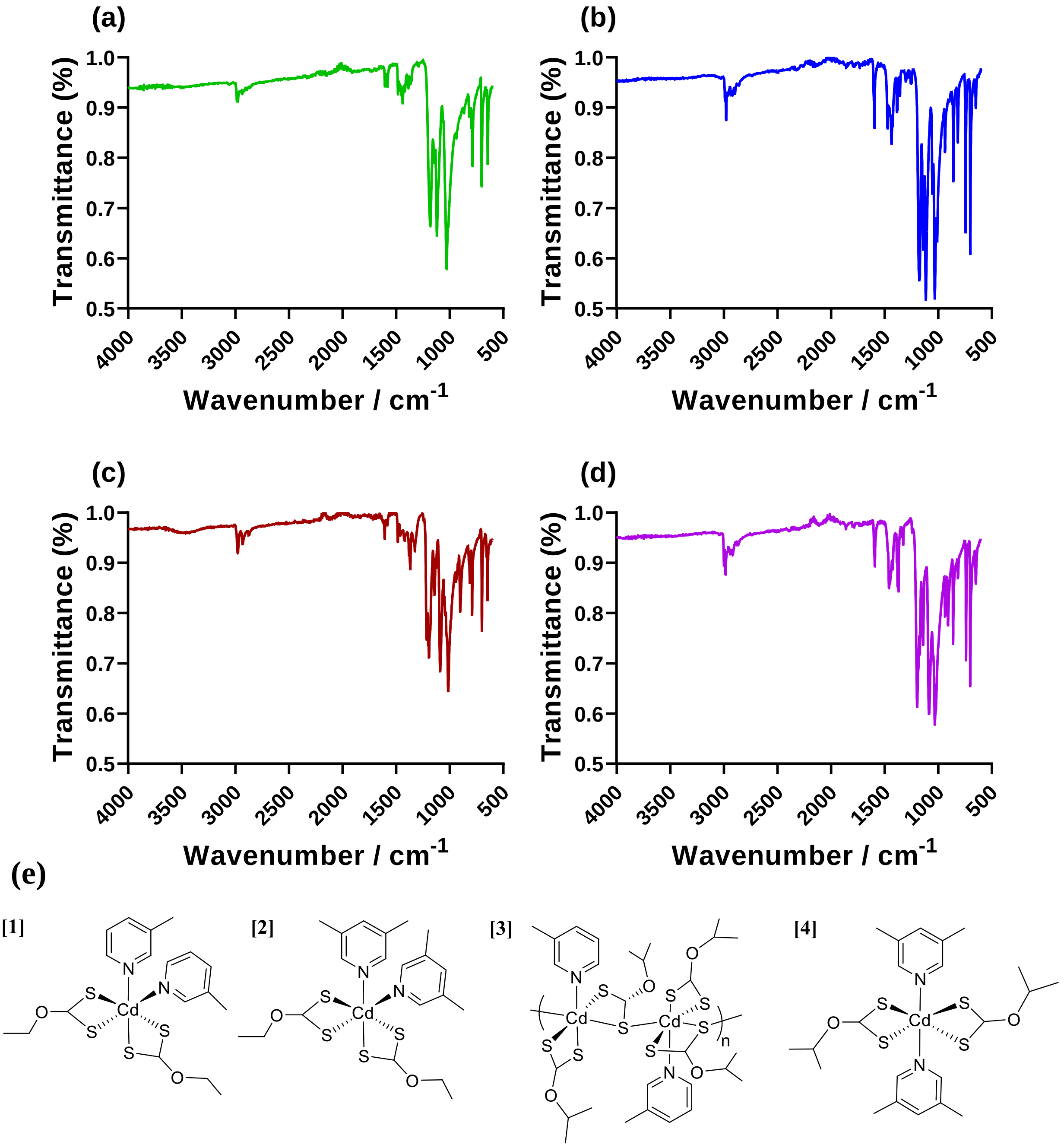

Figure S1 – Figure showing the IR spectra obtained for (a) precursor **[1]**, (b) precursor **[2]**, (c) precursor **[3]** and (d) precursor **[4]**. The structures of the four precursors are shown again in (e) for clarity.

Crystallography of Complex [2], [3] and [4]

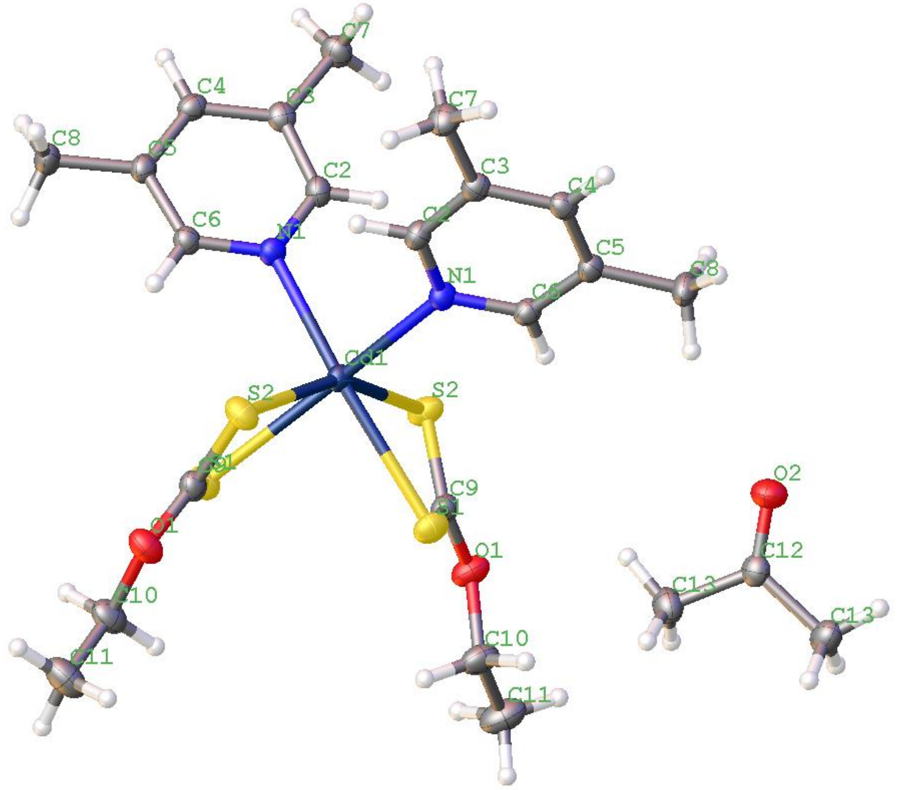

Figure S2 – Crystal Structure of precursor **[2]** with atoms labelled (co-crystalised acetone also included).

Selected bond angles (deg): N(1)–Cd–N(1^1) 90.08(9), N(1)–Cd–S(1) 95.03(5), N(1)–Cd–S(1^1) 163.45(5), N(1)–Cd–S(2) 99.98(5), N(1)–Cd–S(2^1) 96.32(5), S(1)–Cd–S(1^1) 84.47(3), S(2)–Cd–S(2^1) 156.86(3), S(1)–Cd–S(2) 67.303(17), S(1)–Cd–S(2^1) 95.098(19), S(1)–C(9)–S(2) 123.38(14), C(9)–O(1)–C(10) 118.49(19).

Selected bond lengths (Å): Cd–N(1) 2.3310(18), Cd–S(1) 2.7145(6), Cd–S(2) 2.6819(6), S(1)–C(9) 1.685(2), S(2)–C(9) 1.711(2), C(9)–O(1) 1.331(3), O(1)–C(10) 1.465(3).

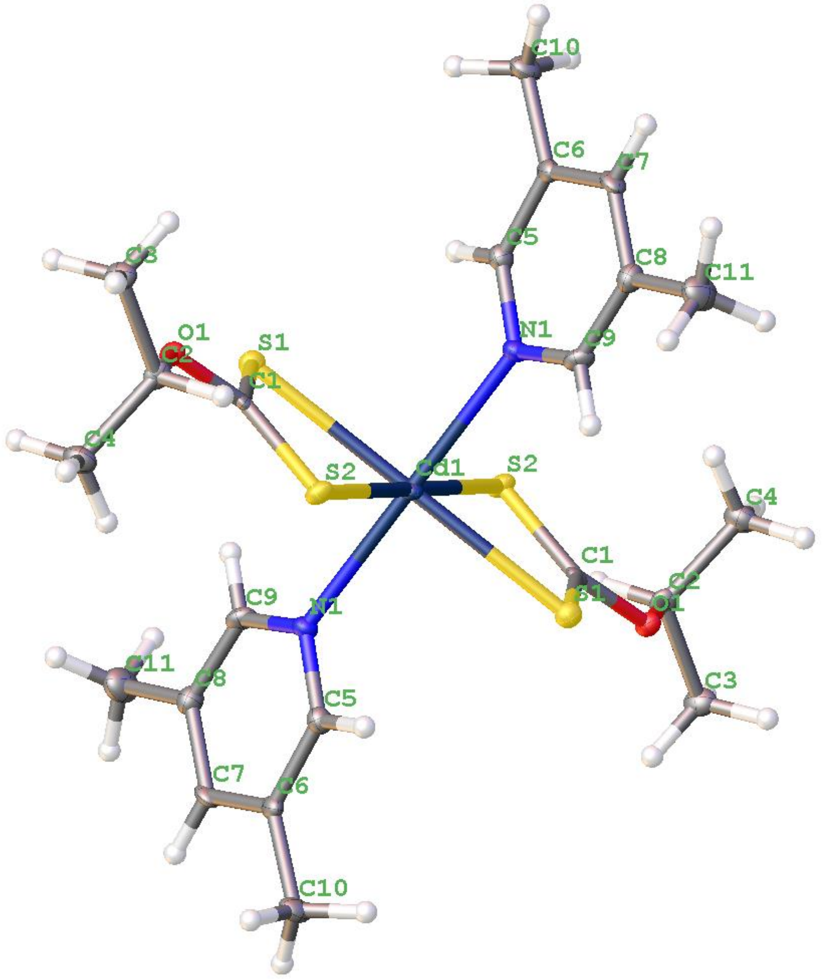

Figure S3 – Crystal structure of CdS precursor complex **[4]** with atoms labelled.

Selected bond angles (deg): N(1)–Cd–N(1^1) 180.0, N(1)–Cd–S(1) 91.73(6), N(1)–Cd–S(1^1) 88.27(6), N(1)–Cd–S(2) 90.54(6), N(1)–Cd–S(2^1) 89.46(6), S(1)–Cd–S(1^1) 180.0, S(2)–Cd–S(2^1) 180.0, S(1)–Cd–S(2) 68.50(2), S(1)–Cd–S(2^1) 111.50(2), S(1)–C(9)–S(2) 124.06(18), C(1)–O(1)–C(2) 121.2(2).

Selected bond lengths (Å): Cd–N(1) 2.414(2), Cd–S(1) 2.6645(8), Cd–S(2) 2.6762(8), S(1)–C(1) 1.703(3), S(2)–C(1) 1.701(3), C(1)–O(1) 1.332(3), O(1)–C(2) 1.474(3).

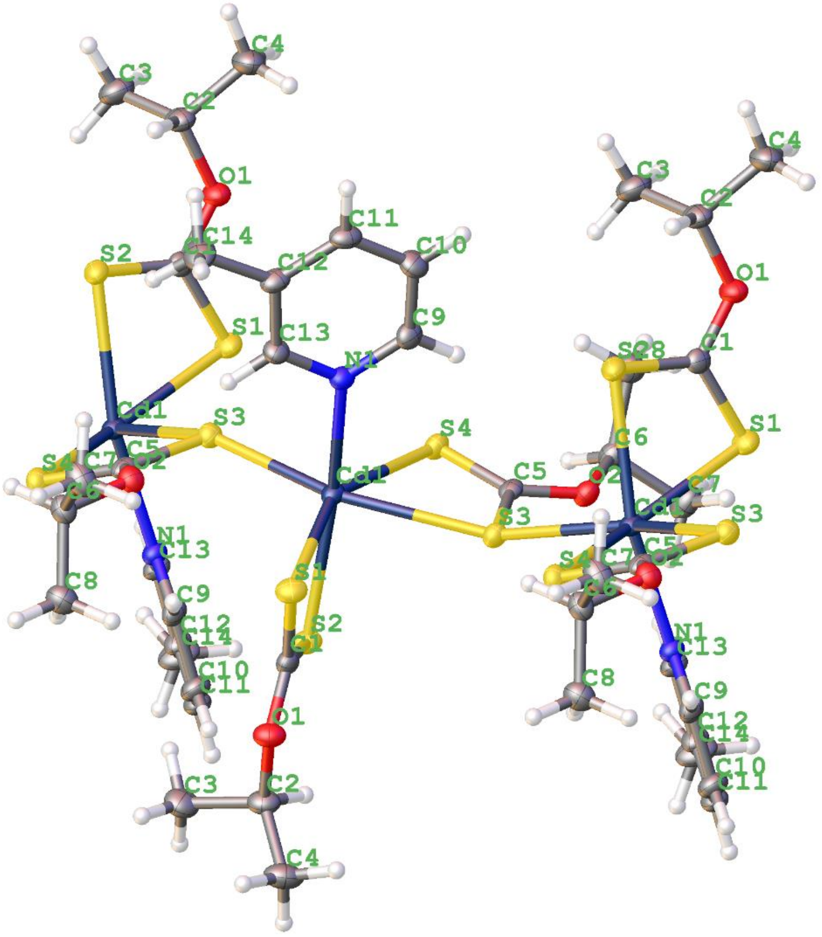

Figure S4 – Crystal structure of CdS precursor complex **[3]** with atoms labelled.

Selected bond angles (deg): Cd(1)–S(3)–Cd(1^2) 152.00(2), N(1)–Cd–S(1) 96.98(6), N(1)–Cd–S(2) 165.14(6), N(1)–Cd–S(3) 93.15(6), N(1)–Cd–S(3^1) 91.75(6), N(1)–Cd–S(4) 95.81(6), S(1)–Cd–S(2) 68.32(2), S(1)–Cd–S(3) 89.01(2), S(1)–Cd–S(3^1) 114.39(2), S(1)–Cd–S(4) 151.49(2), S(2)–Cd–S(3) 88.91(2), S(2)–Cd–S(3^1) 92.50(2), S(2)–Cd–S(4) 98.39(2), S(3)–Cd–S(3^1) 155.294(8), S(3)–Cd–S(4) 64.90(2), S(3^1)–Cd–S(4) 90.52(2), S(1)–C(1)–S(2) 122.53(17), S(3)–C(5)–S(4) 121.97(16), S(1)–C(1)–O(1) 114.6(2), S(2)–C(1)–O(1) 122.9(2), C(1)–O(1)–C(2) 119.5(2), S(3)–C(5)–O(2) 113.95(16), S(4)–C(5)–O(2) 124.1(2).

Selected bond lengths (Å): Cd–N(1) 2.316(2), Cd–S(1) 2.6471(7), Cd–S(2) 2.6607(7), Cd–S(3) 2.8266(7), Cd–S(4) 2.7053(7), S(1)–C(1) 1.706(3), S(2)–C(1) 1.693(3), S(3)–C(5) 1.711(3),

S(4)–C(5) 1.685(3), C(1)–O(1) 1.334(3), C(5)–O(2) 1.329(3), O(1)–C(2) 1.480(3), O(2)–C(5) 1.481(3).

Table of data for crystallography

Table S1 – Table of data for Crystallography measurement parameters, compared to those of **[1]** previously reported.[1]

**Crystal Parameters**	**CdS precursor**			
	**[1]**	**[2]**	**[3]**	**[4]**
Reference	1	This work	This work	This work
Crystal System	Monoclinic	Monoclinic	Monoclinic	Triclinic
Space Group	$P2_1/n$	P2/n	$P2_1/c$	P-1
a / Å	9.8402(3)	11.72560(10)	10.7613(2)	6.9602(9)
b / Å	11.8088(4)	8.72540(10)	10.4210(3)	10.0592(7)
c / Å	19.9554(6)	13.8696(2)	17.5488(3)	10.0744(7)
α / °	90	90	90	82.396(5)
β / °	90.602	100.9700(10)	92.3429(18)	73.326(9)
Γ /°	90	90	90	75.017(8)
Volume / $Å^3$	2318.71(13)	1393.08(3)	1966.33(8)	651.43(11)
Z	4	2	4	1
ρ_{calc} g cm^{-3}	1.55	1.495	1.608	1.522
Temperature / K	150(2)	100.00(13)	99.99(10)	100.15
μ / mm^{-1}	-	9.293	12.904	1.180
F(000)	-	644.0	960.0	305.8
Radiation	-	Cu Kα (λ = 1.54184)	Cu Kα (λ = 1.54184)	Mo Kα (λ = 0.71073)
Reflections collected (independent reflections)	14181 (6903)	16369 (2755)	14964 (4001)	5019 (2968)

Table of data for melting and decomposition by DSC

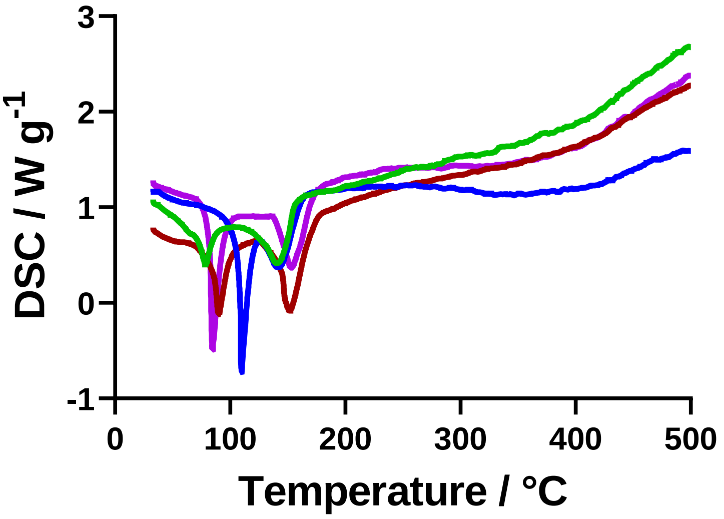

Figure S5 – Figure showing DSC plots of film deposited by precursor **[1]** (in green), **[2]** (in blue), **[3]** (in red) and **[4]** (in purple) with the comparative DSC values.

Table S2 – Table of data for exact melting and decomposition temperatures of the four CdS precursors as determined through DSC analysis. The melting points of these compounds was also independently measured by the traditional method and confirmed the melting point to be within a few degrees of those observed by DSC.

Complex	Melting point (°C)	Decomposition temperature (°C)
**[1]**	79	141
**[2]**	110	142
**[3]**	90	152
**[4]**	84	153

Images of thin films deposited by AACVD and Spin Coating

[1]

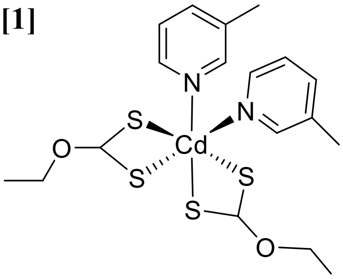

(a)

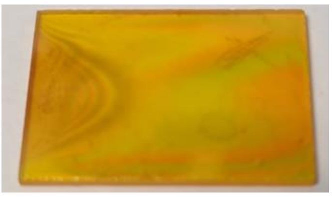

(b)

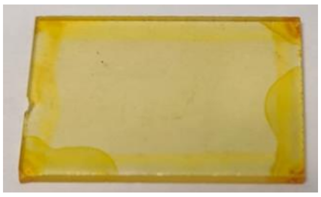

[2]

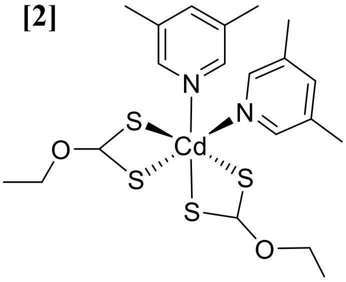

(c)

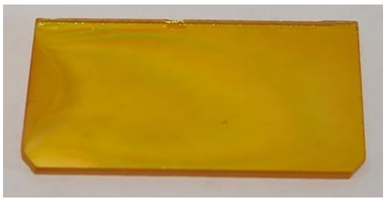

(d)

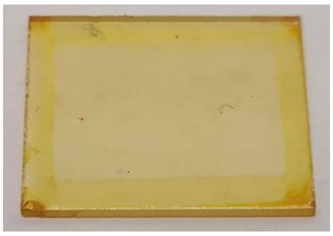

[3]

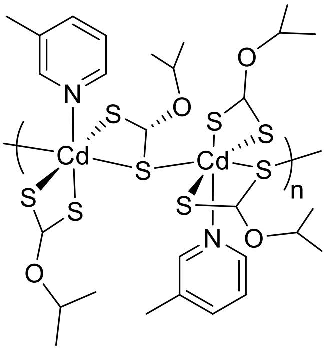

(e)

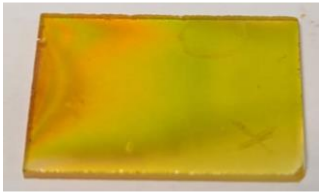

(f)

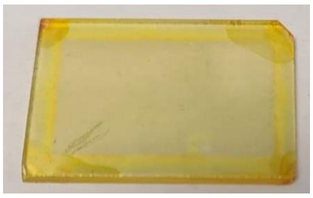

[4]

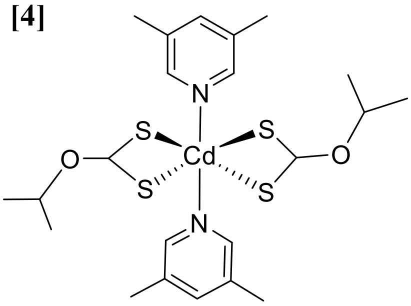

(g)

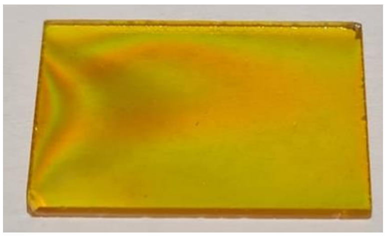

(h)

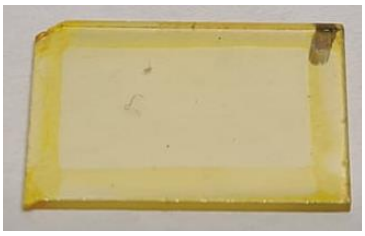

Figure S6 – Figure showing the comparison of deposited CdS films by (left) AACVD and (right) spin coating for complexes (a & b) **[1]**, (c & d) **[2]**, (e & f) **[3]** and (g & h) **[4]**.

The ‘fold’ in the spin coated films (b, d, f & h) is an artifact of the drop-casting method pre-spinning. This is not a thick layer on the corner of the top but a layer on the bottom of the glass slide.

Table of data for Raman Spectroscopy

Table S3 – Table of data for peak position of the Raman Spectra of both AACVD and Spin Coat-deposited CdS films of the four investigated precursors.

Precursor	AACVD Peak positions / cm^{-1}		Spin Coat peak positions / cm^{-1}	
**[1]**	297.7	596.9	299.0	601.7
**[2]**	296.5	592.7	299.0	600.5
**[3]**	297.7	600.5	297.7	596.3
**[4]**	295.2	593.9	298.4	599.3

pXRD patterns of spin coat deposited films

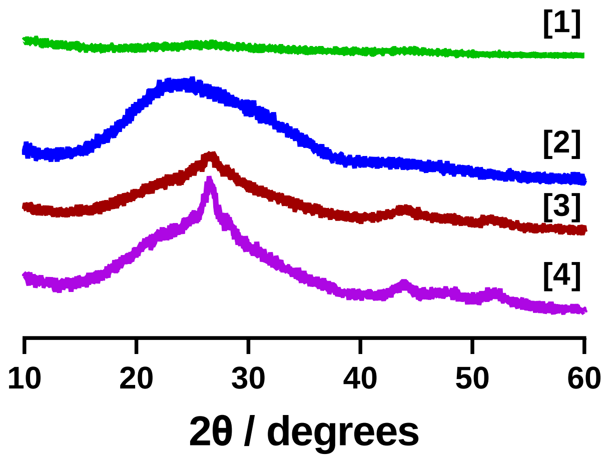

Figure S7 - Figure showing the pXRD patters for the various CdS precursors deposited through AACVD.

Spin coat SEM images

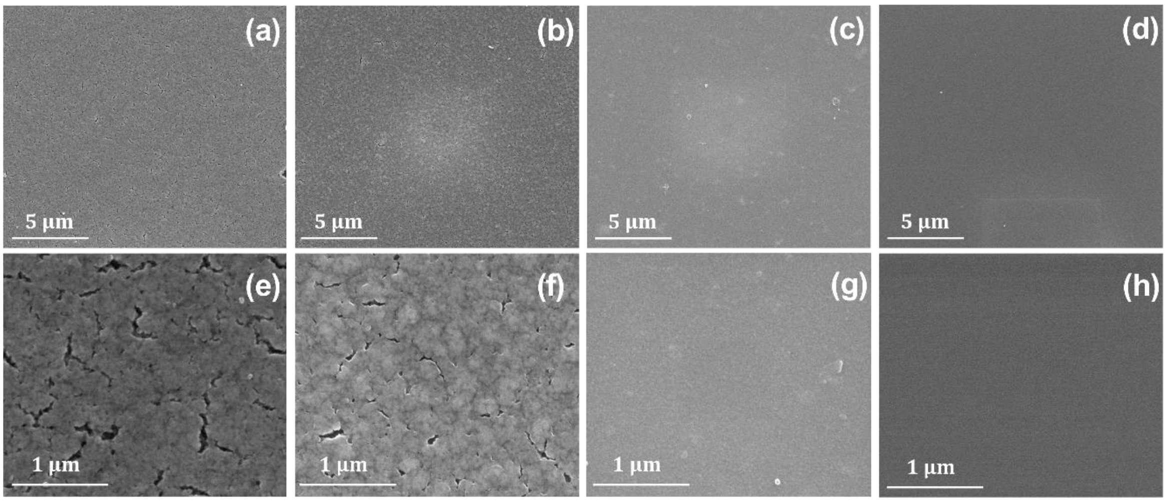

Figure S8 – SEM images of the thin films deposited by spin coating with (a, e) precursor **[1]**, (b, f) precursor **[2]**, (c, g) precursor **[3]** and (d, h) precursor **[4]** parameters the same as those in Figure 4 in the main text.

Table of data for AFM analysis

Table S4 – Table of data for quantitative AFM analysis.

CdS precursor	Measured film thickness / nm	Measured roughness / nm
**AACVD**		
**[1]**	267.4 ± 23.9	61.87 ± 7.7
**[2]**	495.2 ± 71.1	90.25 ± 32.1
**[3]**	219.7 ± 32.8	65.50 ± 30.2
**[4]**	157.9 ± 17.1	42.87 ± 12.4
**Spin Coating**		
**[1]**	86.4 ± 9.8	19.57 ± 8.2
**[2]**	66.1 ± 14.9	20.76 ± 6.0
**[3]**	69.6 ± 14.4	11.84 ± 5.7
**[4]**	64.0 ± 5.0	3.83 ± 0.7

AFM film thickness determination

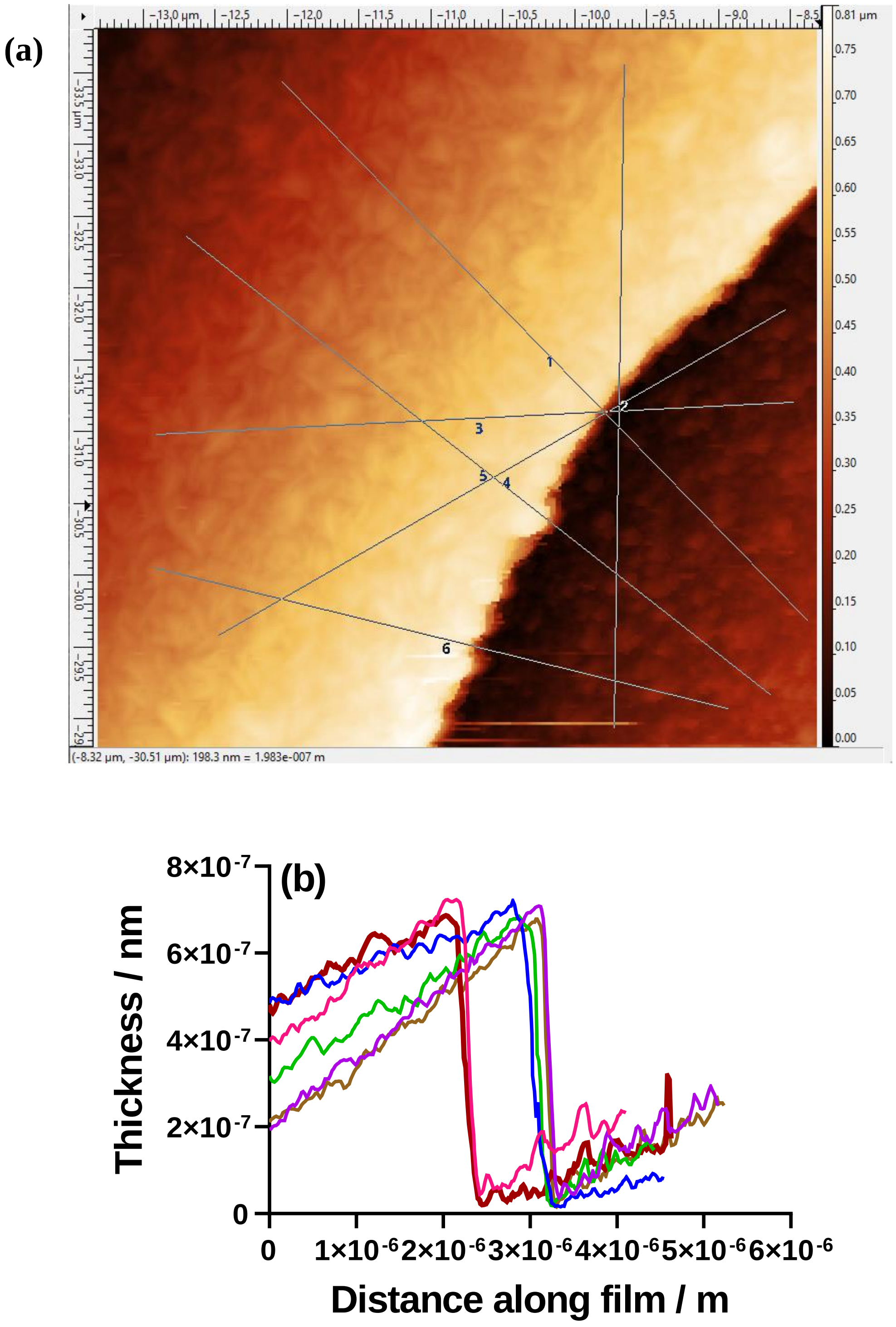

Figure S9 – Figure showing (a) an image of and (b) the randomly selected profile lines for the film deposited from precursor **[2]** deposited by AACVD.

(a)

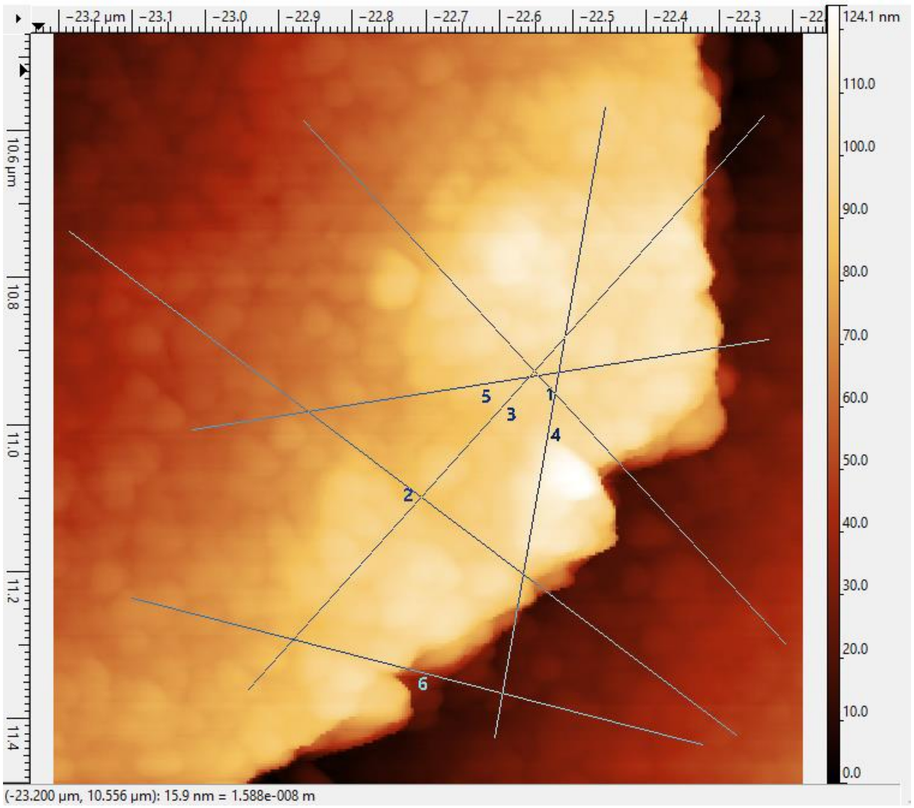

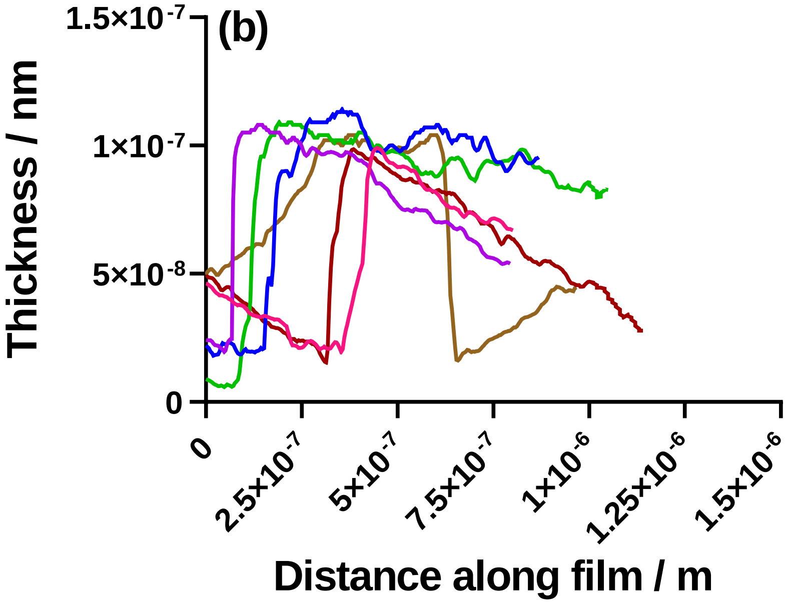

Figure S10 - Figure showing (a) an image of and (b) the randomly selected profile lines for the film deposited from precursor **[3]** deposited by spin coating,

Tauc plots

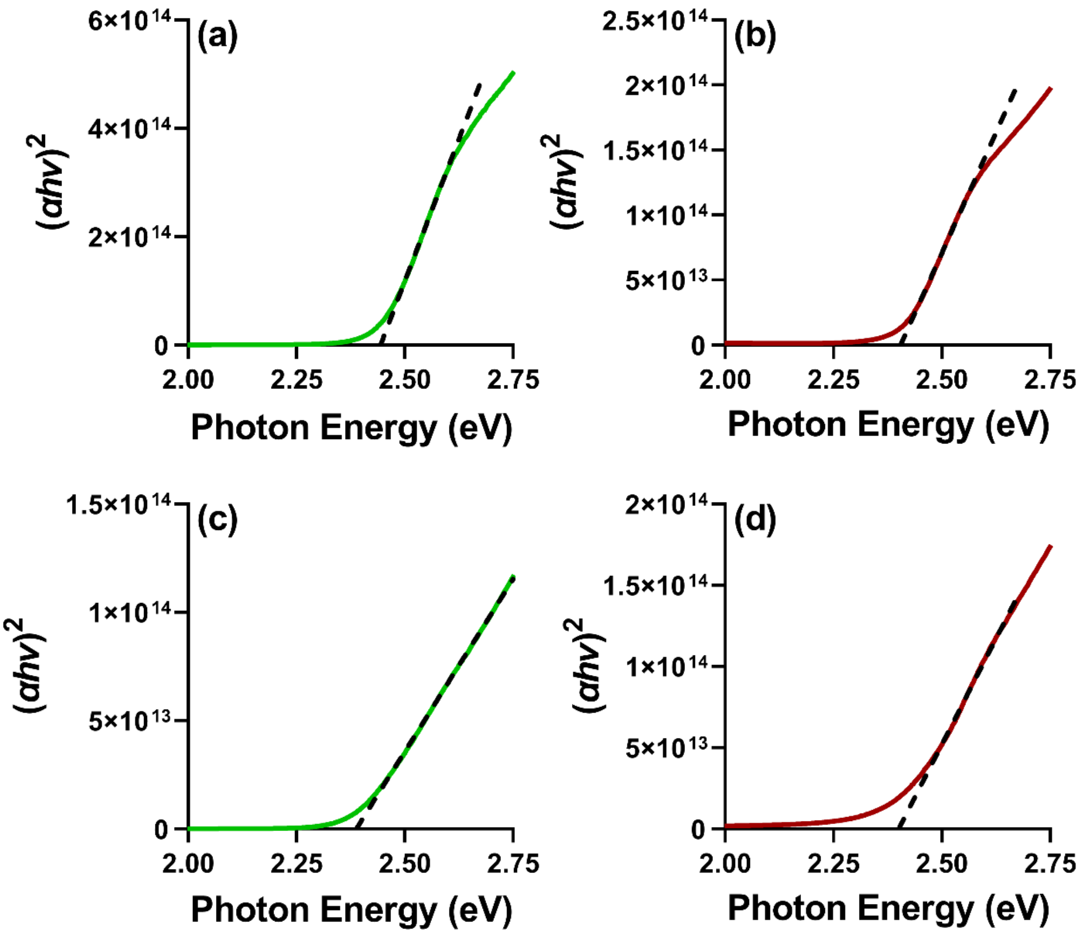

Figure S11 – Figure showing the remaining Tauc plots used in the determination of the band gap for the CdS deposited (a, c) by precursor **[1]** and (b, d) by precursor **[3]** deposited by (a, b) AACVD and (c, d) spin coating.

Table of data from Tauc analysis

Table S5 – Table of data for determined band gap by precursor for both AACVD and spin coat (SC) deposited thin films.

CdS precursor	Band gap energy (eV)	
	AACVD	SC
**[1]**	2.44	2.39
**[2]**	2.48	2.46
**[3]**	2.41	2.40
**[4]**	2.45	2.43

References

1. Buckingham, M. A., Catherall, A. L., Hill, M. S., Johnson, A. L. & Parish, J. D. Aerosol-Assisted Chemical Vapor Deposition of CdS from Xanthate Single Source Precursors. *Cryst. Growth Des.* **17**, 907 (2017).
